# Consensus and controversies on post-acute care decision making and referral to geriatric rehabilitation: A national survey

**DOI:** 10.1016/j.ijnsa.2024.100245

**Published:** 2024-09-24

**Authors:** Aafke J. de Groot, Ewout B. Smit, Dagmar Keizer, Cees M. P. M. Hertogh, Romke van Balen, Johannes C. van der Wouden, Elizabeth M. Wattel

**Affiliations:** a Department of medicine for older people, Amsterdam UMC, Vrije Universiteit Amsterdam, de Boelelaan 1109, 1081 HV, Amsterdam, the Netherlands; b Amsterdam Public Health Institute, Aging & Later Life, de Boelelaan 1117, Amsterdam, the Netherlands; c Careyn, Indusdreef 5, 3564 GV, Utrecht, the Netherlands; d Leiden University Medical Centre, department of Public Health and Primary Care, Leiden, the Netherlands

**Keywords:** Discharge planning, Decision making, Discharge transfer, Geriatric rehabilitation, Patient transfer, Skilled nursing facilities, Subacute care, Triage

## Abstract

**Background:**

Transitioning older hospital patients to the appropriate type of post-acute care has become an urgent clinical issue within the context of changing demographics and limited duration of hospital stay.

**Objective:**

Consensus on assessments that guide post-acute care decision making would benefit potential patients and support cooperation between settings.

**Design:**

A national web-based questionnaire focusing on professional contributions, patient involvement and the use of triage items and measures.

**Participants:**

Hospital and geriatric rehabilitation professionals in the Netherlands participated as respondent groups, representing ‘sending’ and ‘receiving’ professionals.

**Methods:**

A comprehensive questionnaire was used with open, multiple choice and closed questions, exploring in detail how assessment of hospital patients in need of a post-acute care decision was performed. Descriptive statistics were applied together with deductive coding of qualitative data.

**Results:**

A total of 104 hospital liaison nurses (66.7 %) and 52 GR professionals (33.3 %) participated. Respondents were reasonably satisfied with the current triage practice. Hospital liaison nurses valued their operational responsibility for triage. Geriatric rehabilitation professionals wanted active involvement in decision making and deemed hospital paramedic expertise sub-optimally applied. ‘Too little involvement’ of patients and families was felt by 50.0 % of the GR respondents versus 15.5 % of hospital respondents. The importance of half (47.8 %) of the triage items was rated differently between respondent groups. When discussing complex cases between sending and receiving professionals, views were felt as complementary.

**Conclusions:**

Both sending and receiving professionals expressed moderate satisfaction with post-acute care decision making, whereas their views on triage assessments differed according to setting and role. The patients’ voice may be insufficiently heard in triage decisions. Shared expertise and a consensual approach can develop when triage consultation is facilitated by both hospitals and PAC facilities. This study offers ingredients to reach a multi-professional view on post-acute care decision making and referral to geriatric rehabilitation.

**What is already known**

Referral to appropriate post-acute care presents with professional dilemmas and organizational stressors

When patient complexity increases, transition between settings is endangered by information deficits and communicational barriers

The perspectives of ‘referring’ and ‘receiving’ professionals may clarify controversies in triage methods.

Alt-text: Unlabelled box

**What this paper adds**

Hospital professionals focused on rehabilitation needs, geriatric rehabilitation professionals on rehabilitation prognosis.

Multidisciplinary involvement, use of measurement instruments and patient participation in decision making were limited.

The differences and similarities underlying triage practice can serve as a starting point for professional consensus and development of triage guidelines.

Alt-text: Unlabelled box

## 1 Introduction

In older and vulnerable patients hospitalization is frequently accompanied by functional decline, which complicates returning home.(Falk Erhag et al., 2023; Lin et al., 2016) Inspired by the policy of ‘aging in place’ and fortified by restrictions on duration of hospital stay, short-term post-acute care services have developed to support patients in overcoming these problems.(van den Besselaar et al., 2021; GalvinWills and Coffey, 2017; Murmann et al., 2023) In 2019, 15–25 % of hospital discharges of older patients in the US resulted in transfer to a rehabilitation or skilled nursing facility.(Werner and Konetzka, 2018) In view of the growing number of older and very old patients, decision making concerning the transitioning of older hospital patients to the most appropriate type of post-acute care has become an urgent issue with underlying societal and ethical dilemmas that reflect the tensions between individual benefits of post-acute care and societal costs.(Najem et al., 2018)

Post-acute care (PAC) implies a spectrum of short stay medical and skilled nursing services for older and/or frail patients who require a higher level of care than can be provided at home or in long-term care settings. Geriatric rehabilitation (GR) is an often deployed, effective PAC service aimed at rebuilding self-supportiveness in relation to personal and achievable goals, through integrated multidisciplinary care.(Grund et al., 2020) In European countries the availability of GR varied from 0 to 70 beds per 100.000 inhabitants; 53.300 patients received GR in 2019 in the Netherlands. GR can be necessary after a stroke, hip-fracture or other trauma surgery, orthopedic surgery, vascular surgery, such as amputations, and a variety of internal medical illnesses, such as grave episodes of pulmonary and cardiovascular disease or deconditioning after critical illness. The ultimate goal of geriatric rehabilitation is a safe and preferably longstanding return to community living, albeit adapted to irreversible functional loss.

Based on core determinants of rehabilitation, such as functional prognosis, physical needs and mental capacity necessary to partake in treatment, triage procedures were developed. These included ‘motivation to undertake a rehabilitation trajectory’ and ‘readiness for discharge from hospital specialist care’ as building blocks to make proper referral decisions. (GalvinWills and Coffey, 2017; Muscat et al., 2022; Cowley et al., 2021)

In practice however, when patient complexity increases, transitional problems could arise, such as inadequate transfer of medical background information, insufficient knowledge of care in the next setting and communicational barriers.(Lockwood and Mabire, 2020) Both hospitals and GR-facilities recognized the stressing financial aspects that could compromise the quality of patient centered transitional care.(Britton et al., 2017; Lawrence et al., 2020) Based on patient and family interviews in which decision making was evaluated, Burke and colleagues concluded that quality improvement of the decision making process is essential for both patients and professionals. (Burke et al., 2018) In view of the aforementioned challenges in transitional care we wanted to study the clinical methods of decision making concerning referral to geriatric rehabilitation in more detail than previous studies. Which clinical criteria were applied in these decisions and how did professionals cooperate to perform this task?

This study explored the perspectives of both hospital and rehabilitation professionals on methods in post-acute care decision making concerning GR in the Netherlands, aiming to work towards interprofessional consensus on referral to geriatric rehabilitation.

## 2 Methods

### 2.1 Design

This is an explorative cross-sectional survey with quantitative and qualitative data. it. The CROSS reference list was used to report the findings (Sharma et al., 2021)

### 2.2 Questionnaire

**Fig. 1:**
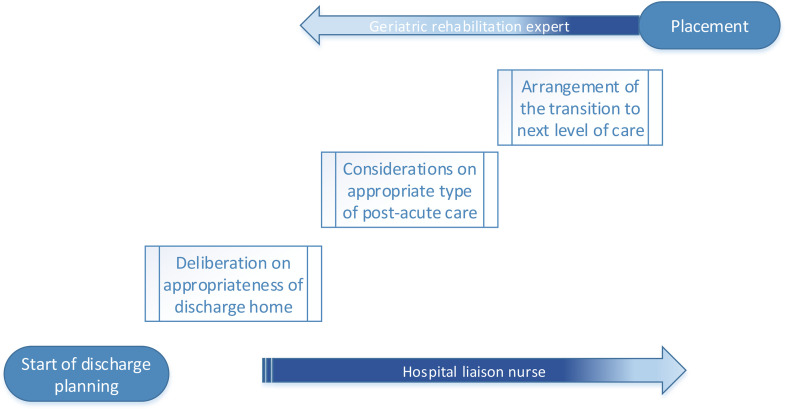
Three steps in post-acute care decision making.

Fig. 1. shows a triage model consisting of three steps in post-acute care decision making, based on the Verenso triage model.(Verenso, 2013) The first step concerns the deliberation that discharge home is expected to be unsafe as further care needs are present. The second step is the assessment of the patient's post-hospital care needs, prognosis and preferences concerning post-acute care, resulting in a post-acute care (PAC) decision. The third step is the actual arrangement of placement in rehabilitative or other post-acute care.

Questions were developed based on international literature concerning referral to GR and national pilot survey's on triage.(Muscat et al., 2022), (de Groot et al., 2022; Bowles et al., 2009; Cowley et al., 2022) An experienced staff liaison nurse commented on the concept versions.

A combination of closed, multiple choice, open and semi open questions was used. Respondents’ views were explored using Likert-scales. Grading of respondents’ satisfaction with aspects of triage was explored using a scale of 1 (very inadequate) to 10 (excellent). Definitions of all concepts were provided to ensure adequate content validity. The final version was piloted in ‘thinking aloud’ telephone sessions, with three liaison nurses and three rehabilitation professionals.

The survey consisted of four parts. The first section explored participants’ background and working experience. The second addressed the involvement of professionals, patients and family in triage decision making. The third captured the triage methods, exploring participants’ considerations on the importance of triage items that underlie decision making. Examples of measurement instruments were given to grade their use . The final section concerned overarching organizational aspects of the triage process. Completion of the questionnaire was expected to take 15 to 20 minutes. See Supplement 1

### 2.3 Recruitment

The survey was distributed among hospital liaison nurses, who coordinate post-acute care placement and among elderly care physicians, specialist nurses and physician assistants working at geriatric rehabilitation departments of nursing facilities. Liaison nurses were contacted in person via the professional association of nurses in the Netherlands (V&VN) (*N* = 200) and via e-mail to the transfer agencies of all Dutch hospitals. A mailing list of transfer agencies was used and the missing hospitals were contacted by telephone. Elderly care physicians, specialized in GR (*N* = 120) were contacted via their professional association (Verenso) and asked to invite GR-nurse specialists and GR-physician assistants to participate. Both liaison nurses and GR professionals were encouraged to invite colleagues working in other hospitals or GR facilities to participate as well. Potential respondents received a reminder two weeks after the first distribution.

### 2.4 Data collection

The survey was distributed using the tool Survalyzer Essential (Zurich, Switzerland) via a web-based link, that could be used from October 13 till November 26, 2020. To guarantee adequate participant selection, the first part of the survey contained a selection procedure for enrollment.

### 2.5 Data analysis

We grouped respondents according to professional background and setting, being either a liaison nurse (LN) in a hospital or a rehabilitation professional in a geriatric rehabilitation (GR) setting.

Nominal variables were presented in frequencies. Ordinal and scale variables, including the Likert items and scales, were presented in frequencies for 3 or 4-point scales and with means for 5-point scales. (Harpe, 2015)Data on triage criteria were listed in domains: ‘somatic’, ‘functional’, ‘psychologic’, ‘social’ and ‘communication’ following the SAMPC model to assess care needs and set goals in medical care for older patients. (Hertogh, 1997)

We used a pragmatic thematic approach in the analysis of the qualitative data concerning professional responsibilities, patient involvement, triage criteria and evaluation of the triage process. Starting with the questions’ subject as a theme, one researcher (XX) categorized the data in subthemes and discussed them with a second researcher (YY) .

Significance was set at 0.05. Independent sample T-test or Mann-Whitney U test were used for intergroup comparison. Statistical analysis was done by IBM SPSS statistics version 28.

### 2.6 Ethics

This survey was exempted from institutional review board approval as it did not involve patients or medical data. The study was performed in line with the principles of the Helsinki declaration (World Medical Association). . Data was stored in compliance with the General Data Protection Regulation. Respondents were informed concerning the aim of the study and the voluntariness of their participation. Answering and returning the questionnaire was considered informed consent to participate in this study.

## 3 Results

### 3.1 Respondents

A total of 156 respondents participated, 102 (67 %) were hospital liaison nurses and 54 (33 %) GR professionals. This response represented 52 % of registered liaison nurses and 47 % of registered GR experts. All respondents were experienced in conducting triage and handled an average of 5–15 cases weekly. Table 1 shows information concerning the participants. The majority of liaison nurses (85.6 %) worked in regional or district hospitals and performed triage for multiple specialisms, such as neurology, orthopedic or trauma surgery and oncology. One third (32.6 %) of the GR respondents was employed in large GR facilities, 40.4 % in facilities with 30–60 GR beds and 26.9 % in smaller facilities. The facilities concerned provided in GR for several diagnostic target groups, in other types of post-acute care and in long term care. Additional background information concerning the GR respondents is in Supplement 2.

**Table 1:** Setting and working experience of respondents.

	Liaison nurses (%) *N* = 104 (66.6)	GR professionals (%) *N* = 52 (33.3)
**Age** (years)		
21–30	4 (3.8)	3 (5.8)
31–40	18 (17.3)	13 (25.0)
41–50	31 (29.8)	13 (25.0)
51–60	39 (37.5)	21 (40.4)
>60	12 (11.5)	2 (3.8)
**Region**		
Urban area	54 (51.9)	14 (26.9)
Smaller city	29 (27.9)	31 (59.6)
Rural	21 (20.2)	7 (13.5)
**Hospital**		
Academic	15 (14.4)	
District	36(34.6)	
Regional	53 (51.0)	
**GR facility**		
>60 places GR		17 (32.6)
30–60 places GR		21 (40.4)
<30 places GR		14 (26.9)
**Work experience** (years)		
< 5	21 (13.5)	12 (23.1)
6–15	26 (16.7)	10 (19.2)
>15	57 (36.5)	30 (57.7)
**Triage experience** (months)		
<6	5 (4.8)	2 (3.8)
6–12	4 (3.8)	4 (2.6)
>12	95 (91.3)	46 (88.5)
**Triage caseload** (cases/week)		
<5	18 (17.3)	8 (15.4)
5–15	62 (59.6)	35 (67.3)
>15	24 (23.1)	9 (17.3)

### 3.2 Professional involvement

Team nurses, residents and liaison nurses contributed most to the first step of decision making, ‘deliberating non-home discharge’. The liaison nurses were considered ‘most influential’ at this point. Although the majority of respondents regarded the impact of all involved professionals ‘adequate’, a quarter (29.5 %) of GR respondents reported ‘insufficient input of the resident’. Other hospital professionals, such as geriatricians, psychiatrists, physiatrists, dieticians, or speech therapists were asked to contribute ‘on demand’. A community nurse, general practitioner or the dementia case-manager, familiar with the patient's situation at home, would occasionally be involved sharing extra background information.

Concerning the assessments and deliberations on appropriate follow-up care liaison nurses contributed most. Almost a third of GR respondents felt that the contribution of physiotherapists (25.0 %) and occupational therapists (31.8 %) on the decision was ‘not enough’ in this step, versus 4.4 % and 5.6 % of liaison nurse respondents. Concerning their own contribution, the opinions of GR respondents and liaison nurses differed as well. More GR respondents (40.9 %) than liaison nurses (7.8 %) felt that the GR specialist had insufficient impact, a minority of liaison nurses (11.1 %) and GR respondents (2.3 %) thought liaison nurses had insufficient impact. If they were involved, the impact of physiatrists and GR-specialists on the decision was felt as ‘substantial’. Table 2 shows the opinions of the respondents about each professionals’ contribution.

**Table 2:** Measure of professional contribution to post-acute care decision making.

	Post-acute care decision making			p- value (Mann-Whitney U test)
Professional contribution	too little N(%)	adequate N(%)	too much N(%)	
Team nurse				0.062
LN (*N* = 90)	13 (14.4)	76 (84.4)	1 (1.1)	
GR (*N* = 44)	12 (27.3)	32 (72.5)	0 (0.0)	
Team manager				0.378
LN	8 (8.9)	79 (87.8)	3 (3.3)	
GR	6 (13.6)	37 (84.1)	1 (2.3)	
Liaison nurse				0.002
LN	10 (11.1)	78 (86.7)	2 (2.2)	
GR	1 (2.3)	36 (81.8)	7 (15.9)	
Physiotherapist				<0.001
LN	4 (4.4)	85 (94.4)	1 (1.1)	
GR	11 (25.0)	33 (75.0)	0 (0.0)	
Occ. Therapist				<0.001
LN	5 (5.6)	84 (93.3)	1 (1.1)	
GR	14 (31.8)	30 (68.2)	0 (0.0)	
Resident				0.010
LN	9 (10.0)	71 (78.9)	10 (11.1)	
GR	13 (29.5)	28 (63.6)	3 (6.8)	
Med. Specialist				0.415
LN	11 (12.2)	72 (80.0)	7 (7.8)	
GR	10.(22.7)	29 (65.9)	5 (11.4)	
Physiatrist				0.089
LN	5 (5.6)	84 (93.3)	1 (1.1)	
GR	4 (9.1)	33 (75.0)	7 (15.9)	
GR expert				<0.001
LN	7 (7.8)	66 (73.3)	17 (18.9)	
GR	18 (40.9)	26 (59.1)	0 (0.0)	

LN = Liaison nurse respondents. GR = Geriatric rehabilitation respondents.

Supplement 3 shows the ratings on professional involvement in triage and qualitative data on professional contributions.

In step 3, transfer to a PAC facility, three quarters of respondents (71.6 % (LN), 69.8 % (GR)) answered that a PAC decision could alter, although they were almost unanimous (90.3 %) that this ‘seldom’ occurred. They attributed such a late change primarily (81 %) to ‘disagreement of the GR facility with the triage decision’. Other reasons were: ‘complex nursing or specialized paramedic care not available in the facility’, ‘long waiting list’ and ‘altered medical situation during waiting days’. Qualitative material yielded additional reasons, such as ‘too early onset of hospital discharge planning’, ‘the patient appears to need long term care’ and ‘the family disagrees with the facility’.

### 3.3 Patients and family

Half of the respondents (55.8 %) thought that patients and families were informed ‘most of the time’ when non-home discharge from hospital was considered, a third (37.8 %) answered ‘always’. This information was shared ‘when discharge was near’ (65 % of respondents) versus 11.2 % ‘at hospital admission’ and 23.9 % who said ‘I don't know’. The mean appraisal for the timing of this communication (1(bad timing) -10 (excellent timing) was 6.4 by liaison nurse respondents and 6.3 by GR professionals.

Liaison nurse and GR respondents felt differently concerning the impact of patients and families in post-acute care decision making. Half (50.0 %) of the GR respondents versus 15.5 % of liaison nurse respondents felt that the patients contributed ‘too little’; most liaison nurse respondents (79.6 %) answered ‘sufficient influence’. They judged the family's contribution ‘sufficient’ (72.3 %) or ‘too much’ (14.9 %), whereas half of GR respondents (51.1 %) rated the family's influence as ‘too little’.

Concerning the contribution of the patient, specifically on choice of placement, liaison nurses responded in 13.6 % ‘not much’, 34.1 % ‘quite some’ and 21.6 % ‘much influence; 29.5 % choose ‘it depends on the situation’. Respondents added ‘patients and families are invited to give their preferences concerning placement options’ and ‘pressure on hospital discharge frequently overrules their choices’.

### 3.4 Triage items

The importance of 11 out of 23 triage items was significantly differently rated by respondent groups. Liaison nurses rated ADL domain items highest. ‘In- hospital mobility loss and/or risk of falling’ and ‘in-hospital functional decline’ were deemed very important by them. Both respondent groups assessed ‘no in-hospital recovery’ and ‘expected recovery’ as very important or decisive. GR respondents rated the psychological domain items highest. In both groups ‘Impaired comprehension of instruction’ was felt important. Societal domain items were higher rated by GR respondents compared to liaison nurses. Table 3 shows the ratings of items.

**Table 3:** Domains and ratings of triage items.

	Liaison nurses *N* = 83 (mean)	GR specialists *N* = 42 (mean)	p-value
**Somatical items**			
Medication >2 times a day	1.99	1.64	0.064
Multiple chronical conditions	3.37	2.69	<0.001*
Incontinence	1.71	1.69	0.897
Pressure ulcer (InH)	2.65	2.52	0.515
Poor nutritional status	2.90	2.60	0.092
Complications (InH)	3.22	2.76	0.005*
Vulnerability	3.30	3.21	0.626
**ADL items**			
ADL limitation or home-care twice a day (PreH)	2.93	3.33	0.014*
Mobility loss and risk of falling (InH)	3.95	2.95	<0.001*
Functional decline (InH)	3.53	3.19	0.046*
Course of previous recovery	2.96	3.12	0.354
No recovery (InH)	3.35	3.14	0.145
Expected recovery (PostH)	4.08	3.74	0.012*
**Social items**			
Living alone	2.67	2.95	0.137
Follow-up care at own request	2.19	3.31	<0.001*
Staircase at home	2.29	2.69	0.043*
**Psychological items**			
Previous psychiatric condition or addiction	3.11	3.50	0.024*
Impaired comprehension of instructions	3.78	3.88	0.463
Impaired awareness of illness	3.25	3.45	0.221
Delirium (InH)	3.55	3.10	0.004*
Anxiety/depression	2.89	2,83	0.707
**Communicational items**			
Severe visual or hearing impairment	3.13	3.05	0.583
**Other items**			
Age	2.73	1.88	<0.001*

ADL = Activities of daily living. InH = In hospital, during hospital stay. PostH = after hospital stay. PreH = before hospital admission. Grading scale: (1)unimportant, (2)somewhat important, (3)important, (4)very important, (5)decisive.

p-value by ANOVA.

In addition other triage items were mentioned, such as ‘expectations and wishes of patient and family’, ‘attitude and motivation concerning GR’, ‘detailed information from community healthcare workers on functional status and participation before hospital admission’, ‘rehabilitation goals’, ‘information from the attending specialist on prognosis’ and ‘patients’ exercise tolerance’.

### 3.5 Measurement instruments

To support post-acute care decisions, instruments for delirium and cognitive status were frequently used, for depression and caregiver burden seldom. Respondents differed significantly in their reports on use of measurement instruments for delirium. See Table 4.

**Table 4:** Use of measurement instruments to support post-acute care decisions.

	Liaison nurses *N* = 83 (%)	GR *N* = 42 (%)	Liaison nurses *N* = 83 (%)	GR *N* = 42 (%)	Liaison nurses *N* = 83 (%)	GR *N* = 42 (%)	Liaison nurses *N* = 83 (%)	GR *N* = 42 (%)	p-value Mann-Whitney U test
	NEVER		OCCASIONALLY		OFTEN		ALWAYS		
Functional status	13 (15.7)	3 (7.1)	30 (36.1)	21 (50.0)	29 (34.9)	10 (23.8)	11 (13.3)	8 (19.0)	0.743
Nutritional status	17 (20.5)	10 (23.8)	26 (31.3)	19 (45.2)	31 (37.3)	7 (16.7)	9 (10.8)	6 (14.3)	0.256
Delirium	0 (0.0)	2 (4.8)	11 (13.3)	17 (40.5)	52 (62.7)	21 (50.0)	20 (24.1)	2 (4.8)	<0.001
Cognitive status	5 (6.0)	3 (7.1)	29 (34.9)	21 (50.0)	42 (50.6)	14 (33.3)	7 (8.4)	4 (9.5)	0.176
Depression	46 (55.4)	17 (40.5)	31 (37.3)	22 (52.4)	6 (7.2)	3 (7.1)	0 (0.0)	0 (0.0)	0.158
Frailty	40 (48.2)	25 (59.5)	27 (32.5)	15 (35.7)	12 (14.5)	2 (4.8)	4 (4.8)	0 (0.0)	0.089
Mobility	39 (47.0)	19 (45.2)	16 (19.3)	18 (42.9)	20 (24.1)	4 (9.5)	8 (9.6)	1 (2.4)	0.294
Caregiver burden	56 (67.5)	28 (66.7)	18 (21.7)	14 (33.3)	9 (10.8)	0 (0.0)	0 (0.0)	0 (0.0)	0.756

Measurement instruments given in questionnaire as an example: Rankin, Katz-ADL, Barthel Index, Short Nutritional Assessment Score, Timed Up and Go, walking speed, Mini Mental State Examination, Montreal Cognitive Assessment, Delirium Observation Scale score, Geriatric Depression Scale, Hospital Anxiety and Depression Scale, Clinical Frailty Scale, Caregiver Strain Index.

Other ADL and mobility instruments respondents mentioned, were instrumental ADL scales, the functional ambulation classification scale (FAC) and the Berg balance scale (BBS). The Identification of seniors at risk score (ISAR) and the Short Dutch Safety Management score (SDSM-vulnerability) were alternative vulnerability measures. Instruments concerning additional triage-relevant clinical domains were the MRC scale for muscle strength and the visual analogous scale for pain (VAS). A majority of GR specialists (58.9 %) versus a minority of liaison nurses (28.9 %) thought that use of measurement instruments to support PAC decision making was too limited.

### 3.6 Cooperation between settings

A GR expert was involved ‘in all triage decisions’ (67.7 %), ‘only in complex cases’(16.1 %) or ‘only in designated patient groups’(8.6 %), such as oncologic or neurologic patients and patients with delirium. Half of respondents (49.6 %) consulted a staff member of the follow-up setting, a minority (21.4 %) consulted an ‘independent’ GR expert. Mean appreciation of the GR contribution was 7.11 (1–10, most insufficient-excellent). Qualitative data showed that a joint deliberation between liaison nurse and GR expert was ‘valuable’ and ‘contributing to shared decision making’.

Case conferences on discharge destination and follow-up care were regularly held in neurology departments (85.4 %) and to a lesser extent in geriatric (52.4 %), internal medicine (51.2 %) and oncology departments (42.7 %). In a quarter (23.9 %) of these conferences, external professionals, such as GR experts were members. Respondents, familiar with these extended case conferences (*N* = 21), were content with them, mean 7.0 (SD 1.3) on a scale from 1 to 10.

### 3.7 Triage responsibility

Three quarters of GR respondents (73.8 %) versus 39.8 % of liaison nurse respondents considered the GR physician or physiatrist responsible for triage decisions. A quarter of liaison nurse respondents (26.5 %) felt this responsibility was theirs. Few respondents (liaison nurses 12.0 %, GR 14.3 %) held the multidisciplinary team or the resident responsible (liaison nurses 9.6 %, GR 4.8 %). Respondents were ‘almost always’ content with the triage decision (liaison nurses: 94.0 %, GR: 97.6 %) and ‘fairly content’ with their own role in triage; liaison nurses graded theirs 7.6 and GR specialists 6.9. on a scale from 1 to 10 (very discontent-very content)

Disagreement on triage decisions was not often reported back to the referring party and structural feedback on percentages of successful GR trajectories was rare. Suggestions for improvements concerned the exchange of information, the use of triage forms, discharge preparation in hospital, communication with patient and family, and ‘time to perform triage carefully’. An overview is in Supplement 4.

## 4 Discussion

This study was performed to gain insight in the multi-professional contributions constituting a referral to geriatric rehabilitation in the Netherlands. We found moderate satisfaction with the triage process and fairly good satisfaction with triage decisions in both hospital and GR professionals. Professional contributions and triage methods were evaluated slightly different by ‘sending’ and ‘receiving’ professionals.

Both liaison nurses and GR professionals felt responsible for the diligence of triage decisions. Liaison nurses felt equipped to take operational responsibility for PAC decision making, whereas GR professionals wished for earlier involvement and opportunities to discuss discharge decisions of complex patients with residents, nurses and paramedic professionals. Taking multi-professional triage decisions in case conferences was valued by both respondent groups. Satisfaction of GR-professionals diminished when involvement occurred only in the last step of triage, ‘placement’, where they had to confirm or review a decision to enable or refuse transition of the patient to the PAC facility. This kind of ‘consultation’ was felt as prompted by the accountability of PAC-facilities for rehabilitation outcome.(Lawrence et al., 2020; Pereira et al., 2023) Professionally, GR consultants would feel a need for detailed medical and paramedical information to deliberate whether a patient could indeed profit from a GR trajectory as the appropriate type of PAC. Concerning this, Wade and colleagues wrote: “whether the person's needs would appropriately fit the profile of rehabilitation care should not be the central triage question”. Instead they plead to ponder “if this service has the best competences to meet this patient's needs compared with other available services”.(Wade, 2022) In a Maltese Delphi study on access criteria for GR, experts felt that clinical complexity and vulnerability of GR-referred patients would often hinder an adequate prediction of rehabilitation outcome.(Muscat et al., 2022) Care needs of geriatric patients do not always fit into ‘appropriate’ post-acute care options and their functional prognosis is seldom unambiguous.

### 4.1 Triage

Between respondent groups, the ratings of triage items differed. Formal criteria for admission to geriatric rehabilitation, such as ‘being a geriatric patient’ characterized by age, multimorbidity or geriatric syndromes were items higher graded by hospital respondents. ‘In hospital functional decline’ and ‘expectation that recovery can be achieved’ was highly rated by both respondent groups. These items determine the actual need for rehabilitation treatment, whereas psychological triage items ‘impaired disease insight’ and ‘impaired comprehension of instructions’ relate to the mental competences necessary to benefit from it. The ADL-item ‘functional level before hospital admission’ that influences goal setting in rehabilitation received high ratings as well.(Wattel et al., 2023; Bouwstra et al., 2017)

Social domain items, higher rated by GR-respondents, concern the likelihood of a patients’ return to the community after the rehabilitation trajectory. ‘Follow-up care at own request’ may indicate an overloaded situation that jeopardizes a return to community living; ‘staircase at home’ relates to the functional status necessary to return home. Different judgement of triage items, can be understood from setting and role of respondents: hospital professionals seem focused on proper placement and GR professionals on outcome of the trajectory. When a patients’ case is discussed, however, these complementary perspectives reinforce each other to the advantage of patient centered decisions.

When referral to GR is considered, careful assessment of preferences and motivation of the older patient is recommended.(de Groot et al., 2022). This survey explored if and when patients and family were informed that post-acute care decisions were to be made. Respondents agreed in a medium appraisal for this timing (6.4) expressing that involvement of patient and family had occurred quite late in the process. More GR than hospital respondents felt that the families’ contribution had not informed the decision. This finding aligns with the study of Gadbois et al. who described that post-acute care placement concerns vulnerable patients and occurs in a hasty fashion. Often information on the options of choice is absent and caregivers select a facility primarily based on its location. (GadboisTyler and Mor, 2017). Although older and vulnerable patients can prefer not to be involved in medical decision making, assessment of their motivation to partake in a rehabilitation trajectory is a core aspect of triage.(Hardicre et al., 2021)

### 4.2 Limitations

The questionnaire was comprehensive and time-consuming for participants. Feasibility and validity of the instrument were not tested. The survey was distributed via professional associations and participants were encouraged to ask their colleagues to participate as well. This may have introduced information bias, as institutions or regions may have been over- or underrepresented.

Finally, this survey was conducted within the boundaries of one country with its healthcare system. Other regulations concerning patients’ access to geriatric rehabilitation may implicate other professional triagist roles.

### 4.3 Strengths

Both ‘sending’ and ‘receiving’ professionals were equally well represented, constituting a representative group of well-informed respondents.

By applying mixed methods of questioning on a large array of triage related subjects, rich data came available. Respondents answered comprehensively to the questions, showing dedication to the subject.

### 4.4 Recommendations

Discussion in multi-professional focus groups can further enhance interpretation of the results. Geriatricians, physiotherapists and occupational therapists that contribute frequently to triage decisions would add valuable insights. The differences and similarities underlying triage practice can serve as a starting point for professional consensus and development of triage guidelines.

Shared expertise on post-acute care decisions develops when triage consultation is facilitated by hospitals and PAC facilities. Exchanging the outcome of geriatric rehabilitation trajectories quarterly between hospital and PAC settings gives common ground to these referrals.

## 5 Conclusion and implications

Liaison nurses and geriatric rehabilitation professionals express moderate satisfaction concerning the triage process and their professional contribution to it. Both feel responsible for adequate triage decisions. Liaison nurses feel equipped for operational responsibility, GR experts wish for more comprehensive involvement in complex cases. They deem multidisciplinary contributions in triage sub-optimal and the voice of patients and families insufficiently heard in the decision. Respondent groups judged triage items differently, but consented that multidisciplinary patient conferences were valuable. Further exploration of similarities and differences between hospital and geriatric rehabilitation professionals can lead the way to consensus concerning triage.

## CRediT authorship contribution statement

**Aafke J. de Groot:** Writing – review & editing, Writing – original draft, Visualization, Formal analysis, Data curation, Conceptualization. **Ewout B. Smit:** Writing – review & editing, Supervision, Methodology, Formal analysis. **Dagmar Keizer:** Methodology, Formal analysis, Data curation. **Cees M. P. M. Hertogh:** Writing – review & editing, Methodology, Conceptualization. **Romke van Balen:** Writing – review & editing, Supervision, Methodology, Conceptualization. **Johannes C. van der Wouden:** Writing – review & editing, Supervision, Methodology, Conceptualization. **Elizabeth M. Wattel:** Writing – review & editing, Supervision, Conceptualization.

## Declaration of competing interest

The authors declare that they have no known competing financial interests or personal relationships that could have appeared to influence the work reported in this paper.

## Appendix Supplementary materials

Image, application 1

Image, application 2

Image, application 3

Image, application 4
