# Supplementary material for: Consensus and controversies on post-acute care decision making and referral to geriatric rehabilitation: A national survey
Source: Int J Nurs Stud Adv. 2024 Sep 24;7:100245. doi: 10.1016/j.ijnsa.2024.100245 (PMC11472103; doi:10.1016/j.ijnsa.2024.100245)
Supplement: Supplementary file 1 [file mmc1.docx]

Supplement 1. Questionnaire.

Composition

The first section explores the participants’ background and working experience.

The second part addresses the involvement of professionals, patients and family in the three phases of triage decision making.

The third section captures the participants’ considerations on the importance of triage items that underlie decision making and the use of measurement instruments for purposes of triage.

In the fourth section of the questionnaire participants were asked to share their views on overarching organizational aspects of the triage process.

I. Background of respondents

(only hospital liaison nurses)

Q3. What type of hospital do you work in?

Q4. In what kind of region is your hospital situated?

Q5. What is your age?

Q6. How many years of practice do you have?

Q7. How long is your experience in performing triage for post-acute care?

Q8. How many triage cases do you handle per week?

Q9. For which specialisms do you perform triage?

(only GR professionals)

Q10. In what kind of region are you working?

Q11. How many beds does your facility have?

Q12. How many beds for geriatric rehabilitation (GR) does your facility have?

Q13. Which GR target groups does your facility offer services for?

Q14. Are there different target groups in the GR category ‘mixed diagnoses’?

Q15. Does your facility offer GR at other locations?

Q16. With how many GR beds?

Q17. For which GR target groups?

Q18. Are there different target groups in the GR category ‘mixed diagnoses’ on this other location?

Q19. What is your age?

Q20. What is your professional background? (i.e. Physician, elderly care specialist, elderly care specialist in training, elderly care specialist-geriatric rehabilitation experts, nurse specialist, physician assistant, other)

Q21. What type of care do you work in?

Q22. How many years of experience do you have?

Q23. How long do you have experience in triage?

Q24. How many triage requests do you handle per week?

Q25. For which target groups do you perform triage?

II. The triage process

Description of triage model consisting of three steps of post-acute care decision making.

The first step concerns the deliberation that discharge home is expected to be unsafe and further care needs are present. The second step is the assessment of the patient’s post-hospital care needs, prognosis and preferences concerning post-acute care, resulting in a post-acute care (PAC) decision. The third step is the actual arrangement of placement in rehabilitative or other post-acute care.

II.1 First step of PAC: deliberation concerning non-home discharge.

Q27. How often are the following hospital professionals (team nurse, team manager, liaison nurse, physiotherapist, occupational therapist, resident, medical specialist) involved in the deliberations on safety of discharge home? (4-point Likert scale: never/sometimes/often/always/unknown). How much impact do these professionals have on this decision? (4-point Likert scale: none/some/quite some/much/unknown).

Q28. Are other professionals involved in this decision?

Q29. Is the patient and his family informed that the necessity of follow-up care is discussed? (Yes always/Yes, most of the time/No, most of the time they are not informed/Unknown/Other…)

Q30. What is your opinion on the contribution of the professionals to this decision? (Too little, sufficient/too much)

Q31. If you answered in Q28 about other professionals, what is your opinion on the contribution of the professionals to this decision?

II.2. Second step: assessments for PAC decision making.

Q32. At which moment is the patient and/or his family involved in PAC-decision making? (at hospital admission/when discharge approaches/this information is not in the request for triage/unknown/other…)

Q33. How do you judge the moment of informing the patient and family on the PAC decision making assessments? (1 not good-10 excellent)

Q34. How often are the following hospital professionals (team nurse, team manager, liaison nurse, physiotherapist, occupational therapist, resident, medical specialist, physiatrist, GR expert) involved in the assessment for PAC decision making? (4-point Likert scale: never/sometimes/often/always/ unknown). How much impact do these professionals have on this decision? (4-point Likert scale: none/some/quite some/much/unknown).

Q35. What is your opinion on the contribution of the professionals to this decision? (Too little, sufficient/too much)

Q36. Are other professionals involved in this decision?

Q37. What is your opinion on the contribution of the professionals to this decision? (Too little, sufficient/too much)

Q38. By which means are you consulted for PAC decision making? (by email, by telephone, in patient conference, in more than one way)

Q39. At what moment are you consulted? (day1-3,day4-6,day7 or later, when the medical specialist treatment is finished, on the day of discharge, other…)

Q40. What is your opinion on the timing of this consultation? Please explain.

Q41. What are the elements of your assessment? (study patient record, discuss case with nurse, discuss case with resident or hospital paramedic, patient is seen, family is seen, different…

Q42. Do you have sufficient information to come to a conclusion on appropriate PAC?

Q43. Do you have sufficient time to come to a conclusion on appropriate PAC?

Q44. What type of involvement of GR experts in PAC decision making occurs most frequently? (case conference, independent GR expert reviews the PAC decision , GR expert of follow-up facility reviews the PAC decision, other….)

Q45. Which PAC decisions are reviewed by a GR expert before admission to a facility? (all GR referrals, only when GR referral is doubtful, only GR special target groups, other…)

Q46. For which target groups is the referral decision reviewed by a GR expert? (stroke, trauma neurology/orthopedic trauma/amputations/COPD/oncology/Parkinsons’disease/Psychogeriatric patients)

Q47. How often is a GR expert acutely consulted by telephone for a triage request? (never/sometimes/often)

Q48. Please describe the last patient this occurred for.

Q49. Are you content with the contribution of the GR expert in PAC decisions? (1-completely discontent-10 most content).

Q50. Please explain your score in Q49.

II.3 Third step: Placement.

Q51. How much influence has a patient on choice of facility for placement?

Q52. What situations or circumstances influence the measure of influence of the patient on placement?

Q53. Hoe content are you with the measure of influence the patient has concerning the choice of facility for placement? (1 very discontent-10 most content)

Q54. Can the PAC decision change in this step?

Q55. What would be the reason for this change?

Q56. How often does the PAC decision change after the patient has been presented for admission to a facility?

Q57. What is the average number of waiting days after a PAC decision is made?

III. Triage items and measurements

Q58. How important are the following items in PAC decision making: Medication >2 times a day, Multiple chronical conditions, Incontinence, pressure ulcers, poor nutritional status, complications, vulnerability, ADL limitation or home-care twice a day, mobility loss and risk of falling, functional decline, course of previous recovery, no recovery in hospital, expected recovery post-hospital, living alone, follow-up care at own or family’s request, staircase at home, previous psychiatric condition or addiction, impaired comprehension of instructions, impaired awareness of illness, delirium, anxiety/depression, severe visual or hearing impairment, age. (5-point Likert scale: not important/slightly important/important/very important/decisive)

Q59. Which other items do you assess to make PAC decisions?

Q60. Which measures do you use for PAC decision making? Rankin, Katz, Barthel Index or other instrument for functional status, SNAQ or other instrument for nutritional status, DOSs or other instrument for delirium, MMSE or MoCA for cognitive status, GDS-15 or HADS for mood complaints, CFS or handgrip strength for frailty, walking speed or Timed Up and Go for mobility, EDIZ or CSI for caregiver burden. (never/sometimes/often/always)

Q61. Do you use other instruments? (which?)

Q62. Are measurement instruments sufficiently applied in PAC decision making?

IV. Cooperation between settings in PAC decision making

(for GR-respondents)

Q63. Are regular multidisciplinary conferences or discharge conferences held for the GR patients that you are involved with concerning triage?

Q64. According to diagnostic groups (GR target groups), for which patients are such conferences held?

(for hospital liaison nurse respondents)

Q65. Are regular discharge conferences held in wards where you are consulted to arrange PAC?

Q66. According to hospital specialism, are such conferences held?

Q67. Are these conferences multidisciplinary?

Q68. Are transmural partners present in these conferences?

Q69. Which disciplines are these transmural partners? (physiatrist/GR expert)

Q70. How content are you with these conferences?(1 very dissatisfied, 10 most content)

Q71. Who is responsible for the PAC decision? (liaison nurse/resident/medical specialist/GR physician or physiatrist, the multidisciplinary team, other..)

(all respondents)

Q72. How often are you content with the PAC decision? (never/sometimes/often/always)

Q73. How content are you with your role in PAC decision making? (1 very dissatisfied, 10 most content)

Q74. Please explain this further.

Q75. What aspects for improvement of triage would you suggest?

Q76. Are results of GR trajectories shared with the referring hospital?

Q77. In what manner is this feedback given?

Q78. How is PAC decision making reimbursed?

Q79. How did this questionnaire reach you?

Q80. Did you miss something in this questionnaire?

Q81. Do you have any remarks on this questionnaire?
