# Supplementary material for: Consensus and controversies on post-acute care decision making and referral to geriatric rehabilitation: A national survey
Source: Int J Nurs Stud Adv. 2024 Sep 24;7:100245. doi: 10.1016/j.ijnsa.2024.100245 (PMC11472103; doi:10.1016/j.ijnsa.2024.100245)
Supplement: Supplementary file 2 [file mmc2.docx]

Supplement 2.

Table S2. Professional background and care settings of GR respondents.

|  | N=156 | N=198 |  |
| --- | --- | --- | --- |
| GR respondents | N=52 (%) |  |  |
| **Professional background** |  |  |  |
| Elderly care physician, GR specialist | 28 (53.8) |  |  |
| Elderly care physician, GR specialist in training | 2 (3.8) |  |  |
| Elderly care physician | 13 (25.0) |  |  |
| Elderly care physician in training | 6 (11.5) |  |  |
| Nurse specialist | 1 (1.9) |  |  |
| Nurse specialist in training | 2 (3.8) |  |  |
|  |  |  |  |
| **N of beds in PAC facility** |  |  |  |
| <100 | 17 (32.7) |  |  |
| 100-200 | 23 (44.2) |  |  |
| >200 | 12 (23.1) |  |  |
| **Care services in PAC facility** |  |  |  |
| Geriatric rehabilitation | 50 (96.2) |  |  |
| Short stay residential care | 29 (55.8) |  |  |
| Long term care | 22 (42.3) |  |  |
| Community geriatric care (incl. day care center) | 14 (25.0) |  |  |
| Other (palliative care, covid-19 unit) | 5 (9.6) |  |  |
| **GR Places** | **In facility**  **N=52** | **In allied facility N=28** |  |
| <15 | 1 (1.9) |  |  |
| 15-30 | 13 (25.0) | 9 (32.1) |  |
| 31-60 | 21 (40.4) | 11 (39.3) |  |
| 61-120 | 15 (28.8) | 7 (25.0) |  |
| >120 | 2 (3.8) | 1 (3.6) |  |
| **Target group GR** |  |  |  |
| Stroke/neurology | 45 (86.5) | 17 (32.7) |  |
| Trauma | 50 (96.2) | 22 (42.3) |  |
| Elective orthopedic surgery | 46 (88.5) | 21 (40.4) |  |
| Amputation | 47 (90.4) | 14 (26.9) |  |
| Other GR diagnoses | 50 (96.2) | 23 (44.2) |  |
| **Target group ‘Other GR diagnoses’** |  | N=23 |  |
| No subdivision/miscellaneous | 21 (42.0) | 11 (21.2) |  |
| COPD | 24 (46.2) | 8 (15.4) |  |
| Cardiology | 16 (30.8) | 3 (5.8) |  |
| Oncology | 20 (38.5) | 4 (7.7) |  |
| Parkinson | 13 (25.0) | 3 (5.8) |  |
| Psychogeriatrics | 9 (17.5) | 3 (5.8) |  |
