# Supplementary material for: Consensus and controversies on post-acute care decision making and referral to geriatric rehabilitation: A national survey
Source: Int J Nurs Stud Adv. 2024 Sep 24;7:100245. doi: 10.1016/j.ijnsa.2024.100245 (PMC11472103; doi:10.1016/j.ijnsa.2024.100245)
Supplement: Supplementary file 3 [file mmc3.docx]

Supplement 3. Professional contributions to PAC decisions

Table S3.1. Mean rating of professional contribution to post-acute care decision making.

|  | Step 1  **Considering non-home discharge** | | Step 2  **Decision on type of**  **post-acute care** | |
| --- | --- | --- | --- | --- |
| **Professional** | Liaison nurses  N=104 | GR professionals  N=52 | Liaison nurses  N=90 | GR professionals  N=44 |
| Team nurse  How often involved  How much impact | 3.61  3.17 | 2.50*  2.23* | 2.83  2.47 | 2.09*  2.02 |
| Liaison nurse  How often involved  How much impact | 3.75  3.94 | 3.77  3.58* | 3.88  3.90 | 3.66  3.59 |
| Physiotherapist  How often involved  How much impact | 3.13  3.47 | 2.19*  2.25* | 3.02  3.31 | 2.20*  2.27* |
| Occ. therapist  How often involved  How much impact | 2.24  2.96 | 1.62*  1.77* | 2.20  2.83 | 1.82*  2.00* |
| Resident  How often involved  How much impact | 3.59  3.26 | 2.73*  2.44* | 3.38  3.21 | 2.61  2.36* |
| Medical specialist  How often involved  How much impact | 2.54  2.79 | 2.50  2.73* | 2.36  2.74 | 2.36  2.55 |
| Physiatrist  How often involved  How much impact | Not involved | Not involved | 2.30  3.83 | 2.39  3.66 |
| GR specialist  How often involved  How much impact | Not involved | Not involved | 2.60  3.62 | 2.75  3.68 |
| Manager  How often involved  How much impact | 1.25  1.34 | 0.94  1.21 | 1.21  1.18 | 0.75*  0.86 |

How often involved? 1=never, 2=sometimes, 3=often, 4=always; How much impact? 1=none, 2= some, 3= quite some, 4=very much; *p<0.05

Table S3.2. Which other professionals contribute to PAC decision making?

| Professional (N) | Contribution | Opinion on contribution |
| --- | --- | --- |
| GR-nurse specialist | GR experience | ‘fine’, ‘sufficient’. |
| Nurse GR facility |  | ‘sufficient’ |
| GR facility placement bureau | Easy accessible for consultation on placement | ‘sufficient’, ‘valued’, ‘in the way between hospital and specialist GR’ |
| Home care or district nurse (4) | Information on patients’ situation before hospital admission. | ‘valued’, ‘important in decision making’. ‘not much influence’ |
| Dementia consulent |  | ‘sufficient’ |
| General practitioner (GP) or assistant of GP (3) | Patient information in complex cases | ‘important, decisive’ |
| Geriatrician (4) | Relevant patient information | ‘valued’; sometimes too much influence. |
| Medical specialist | prognostics | ‘important’. |
| Speech therapist | Professional expertise | ‘sufficient’ |
| Dietician | Professional expertise | ‘sufficient’ |
| Psychologist | Professional expertise | ‘sufficient’ |
| Hospital social worker | Professional expertise | ‘sufficient’ |
| Psychiatrist (2) | Behavioral advice, | ‘sufficient’ |
| Neurology nurse stroke service | Professional expertise | ‘sufficient’ |
| Centralized Office for indication of care(CIZ) (4) | Rejection of indication, difficult communication | ‘negative’ |
