# Supplementary material for: Consensus and controversies on post-acute care decision making and referral to geriatric rehabilitation: A national survey
Source: Int J Nurs Stud Adv. 2024 Sep 24;7:100245. doi: 10.1016/j.ijnsa.2024.100245 (PMC11472103; doi:10.1016/j.ijnsa.2024.100245)
Supplement: Supplementary file 4 [file mmc4.docx]

Supplement 4. Recommendations and remarks on improvement of PAC decision making

|  |  |  |
| --- | --- | --- |
|  | Recommendations | Remarks |
| Patient information | Careful and complete information on patients’ motivation and cognitive problems.  Access to complete medical information.  (More) Use of measurement instruments for triage.  Add measures to triage request.  Make measures of premorbid functioning available.  Describe the prognosis.  Be complete in the information concerning premorbid functioning.  Add care needs and Barthel Index to the file.  Add information of community nurse and homecare.  Be comprehensive in non-somatic information that would complicate recovery.  Deliver more concise and complete information.  Be sure to describe actually correct information.  Paramedical information and the view of paramedics on prognosis is valuable in triage.  Add the medical specialists view on general condition and prognosis of the patient. | STRC patients’ care needs are seldom about recovery.  Incomplete information means more time and worse decisions.  Triage requests differ regarding their form and procedure |
| Assessment | Perform a faster triage process when only advice is requested.  Focus on care indication, not availability of a bed.  Triage by ECP only when in doubt of PAC decision.  Triage by ECP only in complex cases.  Triage by Independent ECP only for complex cases.  ECP is always involved in triage.  Direct conversation between ECP and liaison nurse in case of refusal.  Independent triage direction by liaison nurses.  Allow the liaison nurse to direct 80% of triage cases.  Make the triage process easier by ruling out unnecessary steps.  Use digital communication.  Reduce the influence of the resident in PAC conversations to avoid mistakes.  As a consultant, make sure to see the patients yourself.  Liaison nurse coordinates follow-up care instead of team nurse.  Always involve the ECP as a consulent.  Consult the ECP earlier, before a decision is made.  Involve the ECP more often, earlier and better.  Earlier involvement of ECP.  Start placement procedure even in the absence of ECP’s judgement.  Give the liaison nurse a key position she has an independent opinion.  GR and STRC belong under one indication, indicate the two types of care later, during PAC.  Don’t triage twice.  Involve the ECP in STRC-low complexity triage cases.  Invest triage direction in one person.  Only the ECP of the PAC facility conducts triage.  Skip triage altogether until after PAC-admission a case conference is held. | ECP’s are focused on their own facility.  Other ECP repeats triage procedure.  ‘Motivation’ hard to capture in patients that are tired of living.  Opinions of liaison nurse and facility differ.  GR is not always necessary despite paramedic treatment. |
| Forms and Electronic health records | Use one and the same electronic system.  Make a triage form that is complete and easy to fill out.  Review the structure of the transitional file.  Complete anamneses, patients’ history, in EHR.  Fill in the form to consult a liaison nurse completely.  Consult a liaison nurse sooner. | Forms, EHR’s are confusing and unclear. |
| Discharge planning | Start triage at hospital admission, work discharge oriented.  Improve the quality of the nurse intervention plan.  Take patients’ history carefully and professionally as a nurse.  Liaison nurse and resident must discuss and agree on PAC options.  Residents and nurses are well informed about PAC options.  Consult the liaison nurse sooner, as early as possible.  Work discharge oriented, discuss and adjust expectations, prepare patient and family. | Resident advises for impossible PAC options, and nurses don’t know.  Medical stability not yet reached or uncertain |
| Communication with patient and family | Inform patients and families completely and as early as possible.  Discuss patients’ preferences before the case conference.  Explore patients’ care needs.  Tell patient that the focus of the hospital stay is treatment, not a change of living situation.  Explore the expectations of patient and family.  Inform family before discharge planning starts.  Involve patient and family in decision making.  Inform patient and family on follow-up care and PAC facility.  Attending physician communicates well with patient and family.  ECP can visit patient and family before transition to PAC facility. |  |
| Communication with PAC facility | Early alignment between settings.  Discuss complex cases with ECP. | Questions between parties go back and forth. (time consuming) |
| Time | Response of ECP within 2 hours of triage request.  Enough ECP’s to answer triage requests in time.  Enough time for liaison nurses to take the lead in triage.  Carrying out triage direction and responsibility means that expansion of working hours is needed.  Time to follow the illness trajectory of patients.  Patients with many ‘bed days’ demand more time for follow-up. | Too much text in transitional records  Time pressure obstructs cooperation and communication. |
| Case conferences | Communicate complex cases  Use measurements that support decision making  ECP should participate more often in hospital case conferences |  |
| Cooperation | Consult the hospital physiatrist.  Liaison nurses must partake more in case conferences.  Cooperation with ‘next step’ facilities.  Cooperation in regional networks of PAC.  Regularly scheduled moments for triage consultation.  Hospital and PAC facilities agree on ECP’s role.  More feedback between settings on outcome of PAC trajectories.  Overview of empty PAC beds. | ECP’s hard to contact.  Hospital specialists hard to contact. |
| Guidelines | Clear, transparent triage guidelines.  Universal score system for triage.  ECP’s have a shared view and judgement concerning triage.  Healthcare regulations are correctly applied. | Barthel Index criteria are differently applied. |
| Competency | All ECP’s are competent to conduct triage.  Hospitalists know how to conduct triage.  Hospitalists understand what GR is.  More triage knowledge for hospital nurses and physicians. |  |
| Healthcare policy | More beds for STRC.  Enough PAC beds.  No contrary interests in triage.  More beds for GR patients with dementia. | Financial reasons for refusal of patients. |

ECP: elderly care physicians. EHR: electronic health record. GR: geriatric rehabilitation. PAC: post-acute care. STRC: short term residential care.
